# Identification of multiple genes encoding SnRK1 subunits in potato tuber

**DOI:** 10.1371/journal.pone.0200321

**Published:** 2018-07-06

**Authors:** Yongzhong Zhang, Binquan Huang

**Affiliations:** 1 College of Agronomic sciences, Shandong Agricultural University, Taian, China; 2 University of Lille, CNRS, UMR, Lille, France; Shanghai Institutes for Biological Sciences, CHINA

## Abstract

**Background:**

Many studies have proven the importance of SnRK1 in the regulation of carbohydrate metabolism and plant development. Compared to Arabidopsis, much less is known about SnRK1 complexes in crop plants, and therefore, more work needs to be done to identify SnRK1 genes and to investigate their function in crop plants.

**Methods:**

In this study we identified five SnRK1-related genes in potato by analyzing the potato genome through BLAST, which encode one α-subunit isoform (stKIN), two β-subunit isoforms (stKINβ1 and stKINβ2) and two γ-subunit isoforms (stKINγ and stKINβγ). To investigate the functions of SnRK1 in the tuber development of potato, we further made overexpression and RNAi transgenic plants of these five genes. Based on these overexpression transgenic plants, the Fast protein liquid chromatography (FPLC) were employed to purify SnRK1 complexes, which were tracked by western-blot.

**Results:**

Experiments *in vivo* and *in vitro* showed that these five proteins in potato are functional SNF1/AMPK/SnRK1-related proteins. The SnRK1 activity decreased by 60% in the RNAi transgenic lines of *stKIN*; the starch content increased by 25% in the overexpression transgenic lines of *stKIN*, compared to that in the wild-type lines, whereas there is no significant difference in SnRK1 activity and starch content in the RNAi transgenic or overexpression lines of *stKINβ1*, *stKINβ2*, *stKINγ* and *stKINβγ*. In addition, we found that a few different SnRK1 complexes are present in potato by partially purifying SnRK1 complexes from these overexpression transgenic plants.

**Conclusions:**

Five functional SnRK1-related genes were identified in potato, including three novel genes, which encode one α-subunit isoform (stKIN), two β-subunit isoforms (stKINβ1 and stKINβ2) and two γ-subunit isoforms (stKINγ and stKINβγ). We found that a few SnRK1 related genes are present in potato tuber, which form several different SnRK1 isoenzymes. We found that stKIN is the primary α subunit of SnRK1 in potato tuber and plays important roles in the development of potato tubers.

## Introduction

Sucrose non-fermenting-1-related protein kinase (SnRK1) is a member of the SNF1 protein kinase family in higher plants, which is considered a central regulator of plant growth and development [1–3]. Similar to the SNF1 and AMPK, plant SnRK1 is proposed to form an active heterotrimeric complex with β and γ regulatory subunits. In this complex, the α subunit is responsible for catalytic activity; the β subunit has both kinase interaction sequence (KIS) and association with SNF1 complex (ASC) domains and is therefore generally considered a scaffold, which is responsible for regulatory activity. However, the SnRK1 complex shows some structural diversity and has evolved unique regulatory subunits, such as a truncated β subunit that lacks the KIS domain and an atypical γ subunit with a KIS domain sequence specific to the *β* subunit fused to its N-terminus, which is designated a βγ subunit [4–6]. These plant-specific regulatory subunits might have arisen to perform plant-specific functions.

Each subunit of AMPK and SnRK1 is encoded by distinct genes. AMPK has two α-type subunits (α_1_, α_2_), two β-type subunits (β_1_, β_2_) and three γ-type subunits (γ_1_, γ_2_, γ_3_). In plants, most research has focused on Arabidopsis, which has two α-type SnRK1 subunits (α_1_, α_2_), three β-type subunits (β_1_, β_2,_ β_3_) and two γ-type subunits (γ_1,_ βγ_1_). A previous study has shown that these subunits can form different SnRK1 isoenzymes [6]_._ Compared with those in Arabidopsis, much less is known about SnRK1 complexes in crop plants. A few studies have shown that SnRK1 plays important roles in the regulation of metabolism in crop storage organ and its development, which indicated the value of SnRK1 in the improvement of crop yield and quality. In potato tuber, overexpression (OX) of SnRK1 caused the activity of ADP-glucose pyrophosphorylase (AGPase) to increase, leading to a significant increase in starch content [7]. In barley, SnRK1 is thought to regulate storage product accumulation during grain filling [8]. In wheat, SnRK1 regulates seed filling and development via Trehalose 6-phosphate (T6P) [9], and in maize, two SnRK1-related genes that encode βγ proteins were shown to have an important role in maize kernel development [10]. Overall, compared to Arabidopsis, more work needs to be done to identify SnRK1 genes and to investigate their function in crop plants.

Hence, in this study, we identified five SnRK1-complex-related genes in potato tuber: *stKIN*, *stKINβ1*, *stKINβ2*, *stKINγ* and *stKINβγ*, and further made the OX and RNAi knockdown (KD) transgenic plants to examine the function of these SnRK1-related genes. The *stKIN*-related transformants showed strong phenotypes with significantly altered SnRK1 activity and starch content, while no significant phenotypes were found in the OX or KD transformants of *stKINβ1*, *stKINβ2*, *stKINγ* or *stKINβγ*. Furthermore, we partially purified SnRK1 complexes from these overexpression transgenic plants and found that a few different SnRK1 complexes are present in potato tuber.

## Materials and methods

### Plant material and transformation

Nodal segments of potato cv. Désirée were grown in 50 ml tubes with standard medium MS30 (MS 4.405 g, Sucrose 30 g, agar 8 g, pH 5.7). The transgenic potato plants were generated by using agrobacteria-mediated transformation [11]. The transgenic potato plantlets were transferred into pots and grown in greenhouse at 20°C under a16-h light: 8-h dark regime.

### Development of overexpression and knockdown transgenic plants

To investigate the functions of these five SnRK1-related genes during the tuber filling in vivo, we made both OX and KD transgenic plants for *stKIN*, *stKINβ1*, *stKINβ2*, *stKINγ* and *stKINβγ*. For OX lines, the full-length cDNAs encoding potato stKIN, stKINβ1, stKINβ2, stKINγ and stKINβγ were cloned into the Gateway entry vector pDONR201, and then were further introduced into the pMDC32 vector. The following primers were used for full length amplification:

*stKIN*-F 5’-AAAAAGCAGGCTCCATGAGTTCCAGAGGTGGTGGAATTG-3’

*stKIN*-R 5’-AGAAAGCTGGGTTTGCACTGAGAAATAATGAATG-3’

*stKINβ2*-F 5’-AAAAAGCAGGCTCCATGGGGAATGTGAGTGGG-3’

*stKINβ2*-R 5’-AGAAAGCTGGGTTCTTTTTTAAGTTTTTCAA-3’

*stKINβ1*-F 5’-AAAAAGCAGGCTCCATGGGAAATGCGAACGCCAG-3’

*stKINβ1*-R 5’-AGAAAGCTGGGTTCCTCTTCAGTGGCTTGTA-3’

*stKINγ*-F 5’-AAAAAGCAGGCTCCATGGTGATGGAAGAAGGATG

*stKINγ*-R 5’-AGAAAGCTGGGTTAACCCTGCTATTTGCTGG

*stKINβγ* -F 5’-AAAAAGCAGGCTCCATGTTTGGGTCTGGTAGTG-3’

*stKINβγ* -R 5’-AGAAAGCTGGGTTGCAGCCGAGCAAGAACC-3’

The *stKIN*, *stKINβ1*, *stKINβ2*, *stKINγ* and *stKINβγ* sequences were fused with a HA tag sequence at the C terminus so that the transformants would express proteins fused with the HA tag. For KD lines, a specific fragment of approximately 250 bp from each gene was cloned into pDONR201 for RNAi vector construction, and the fragment of each gene was integrated into the pHellsgate12 RNAi vector. The following primers were used for specific fragments amplification:

*stKIN*-F 5’-AAAAAGCAGGCTCCGCTAGGCAACATCTTAAAAAG-3’,

*stKIN*-R 5’-AGAAAGCTGGGTTTGCACTGAGAAATAATGA-3’;

*stKINβ1*-F 5’-AAAAAGCAGGCTCCATGGGAAATGCGAACGCCAGAG-3’;

*stKINβ1*-R 5’-AGAAAGCTGGGTTACTCTCTAGTGAATGATCAGAGGC-3’;

*stKINβ2*-F 5’-AAAAAGCAGGCTCCATGGGGAATGTGAGTGGGAAGAAG-3’;

*stKINβ2*-R 5’-AGAAAGCTGGGTTCTTCCAGCCATCCCATGATCC-3’;

*stKINγ*-F 5’- AAAAAGCAGGCTCCGTATGGATCCTCCATCAGTCGG-3’;

*stKINγ* -R 5’-AGAAAGCTGGGTTCACGGGAATACTCTTCATCTTGTA-3’;

*stKINβγ* -F 5’-AAAAAGCAGGCTCCAGGGAGTCAGATGCTATTCCTGAACTG-3’;

*stKINβγ* -R 5’-AGAAAGCTGGGTTCCCTTTCCAAGCAGATATAGAGTG-3’.

These constructions were transformed into Agrobacteria AGL1 strain, which was then infected potato callus. Transformations with the empty vector were used as negative controls. Transgenic potato lines were identified by gene-specific and vector-specific primers, and the positive lines were transferred to soil. After genotyping, total RNA was extracted from the tubers of positive transformants to determine the transcript level. The three transformants for each gene with the largest transcript level changes were used for analysis.

### Protein extraction and purification

All the following procedures were carried out at 4°C, except where otherwise stated. Fresh potato tubers at 48, 52, 56 and 60 days after planting were collected from the greenhouse, cleaned and sliced into prechilled protein extraction buffer (50 mM Tris-acetate, pH 7.5, 10 mM EDTA, 2.5 mM dithiothreitol (DTT), 0.1 mM PMSF, 1x protease inhibitor cocktail (P9599, sigma)) and homogenized with a homogenizer (Polytron homogenizer PT 10–35). The homogenate was then centrifuged at 35,000 rpm for 30 minutes at 4°C; the supernatant was further filtered by flowing through a 0.45 μm filter and then transferred into new tubes for use or stored at -80°C until use. Each filtered sample was loaded on an anion-exchange column equilibrated with buffer A (50 mM Tris-acetate, pH 7.5, 10 mM EDTA, 2.5 mM DTT) at a flow rate of 0.5 ml/min. The AEC column connected with an AKTA FPLC system and was washed by buffer A, followed by a gradient of 0% to 60% buffer B (50 mM Tris-acetate, pH 7.5, 10 mM EDTA, 2.5 mM DTT, 0.5 M NaCl) over 40 minutes. Each fraction was 0.5 ml. These fractions were monitored by SnRK1 assays. We pooled all active AEC fractions and concentrated them into 500 μl. The concentrated samples were applied to a Superdex 10/300 GL column (GE Healthcare, catalogue no. 17-5175-01) connected with an AKTA FPLC system. The column was equilibrated and run in buffer A containing 150 mM NaCl. The flow rate was 0.5 ml/min, and the fraction size was 0.5 ml. A molecular weight marker (Bio-Rad, catalogue no. 151–1901) was loaded onto the gel permeation chromatography (GPC) column, and the standard proteins were separated using the same conditions.

### Co-immunoprecipitation

Developing tubers from the OX lines of *stKIN*, *stKINβ1*, *stKINβ2*, *stKINγ* and *stKINβγ* were harvested from the greenhouse, cleaned and sliced into the prechilled immunoprecipitation (IP) buffer containing 25 mM Tris-HCl, pH7.5, 150 mM NaCl, 0.5% Triton-X-100, and 1% protease inhibitor, and the mixture was homogenized with a homogenizer. The homogenate was then centrifuged at 35,000 rpm for 30 minutes at 4°C, and the supernatant was further filtered by flowing through a 0.45 mm filter. The protein extract was then diluted to a concentration of 1–2 μg/μl in IP buffer. The over-expressed stKIN, stKINβ1, stKINβ2, stKINγ and stKINβγ were purified by using Thermo Scientific ^TM^ Pierce ^TM^ HA-Tag IP/Co-IP Kit (Catalogue No. PI26180).

### SnRK1 assays

The SnRK1 assay followed Zhang et al. (2009) [12]. SnRK1 was assayed in 25 *μ*L in duplicate in microtiter plate wells at 30°C. Assay medium was 40 mm HEPES-NaOH, pH 7.5, 5 mm MgCl2, 200 *μ*m ATP containing 12.5 kBq [*γ*-33P]ATP (GE Healthcare), 200 *μ*m AMARA peptide (Ala-Met-Ala-Arg-Ala-Ala-Ser-Ala-Ala-Ala-Leu-Ala-Arg-Arg-Arg), 5 mm dithiothreitol, 1 *μ*m okadaic acid, 1 *μ*m pepstatin A, 10 *μ*m E64, and 7 *μ*m chymostatin. The detailed procedures of the SnRK1 assay exactly followed Hastie et al. (2006) [13]

### Quantitative analysis of starch and soluble carbohydrate content of tubers

Tubers were harvested at 60 days after growing and the cleaned tubers were sliced by scissors and then homogenized in buffer (50 mM Tris-acetate, pH 7, 5 mM MgCl_2_, and 0.5 mM EDTA, 2.5 mM DTT, 0.1 mM PMSF, 1X protease inhibitor cocktail) with a homogenizer. Subsequently, three duplicate 1 ml samples were collected from the tuber homogenate; two of them were used for determining starch content, and the third sample of the crude homogenate was dried at 80°C to determine the dry weight of the tissue analyzed. The starch pellet in each duplicate sample was washed twice by 80% (v/v) ethanol, and then the starch pellet were dissolved in dimethyl sulfoxide. The amount of hydrolyzed starch was qualified by using amyloglucosidase (Megazyme; catalogue no. 9032-08-0) and GOPOD (Megazyme; catalogue no. K-GLUC) to perform a colorimetric assay.

## Result

### Identification of novel SnRK1 related proteins in potato

To identify potato SnRK1-related genes, we blasted the published sequences of SNF1/AMPK/SnRK1-related genes (SNF1, NM_001180785.3; AMPK, NM_019142.2; and SnRK1, M74113.1, AT3G01090, AT3G29160 AJ132315, AJ132316 and NM_100773.4, AT2G28060) against the potato genome, and then used the NCBI Conserved Domain Database (CDD) to perform domain analyses of the identified protein sequences. Finally, we identified five proteins containing conserved SNF1/AMPK/SnRK1-related domains with high confidence, including one α-subunit isoform, two β-subunit isoforms and two γ-subunit isoforms. We named the identified sequences according to the nomenclature of SnRK1 complex: the sequence PGSC0003DMG400024561 encodes a protein with a conserved kinase domain and was designated *stKIN*; PGSC0003DMG400004655 and PGSC0003DMG400017398 encode proteins with conserved KIS and ASC domains and were designated *stKINβ1* and *stKINβ2*, respectively; The PGSC0003DMG400015241 encodes a protein with CBS domain and was designed *stKINγ*; PGSC0003DMG402024742 encodes a protein with both *β-* and *γ-*subunits-related domains, and were designated *stKINβγ*. Of these five genes, the *stKIN* and stKINβ1 were investigated before, showing that they are components of SnRK1 complex in potato [14–17].

### StKIN, stKINβ1, stKINβ2, stKINγ and stKINβγ are functional SnRK1-related proteins

**Fig 1:**
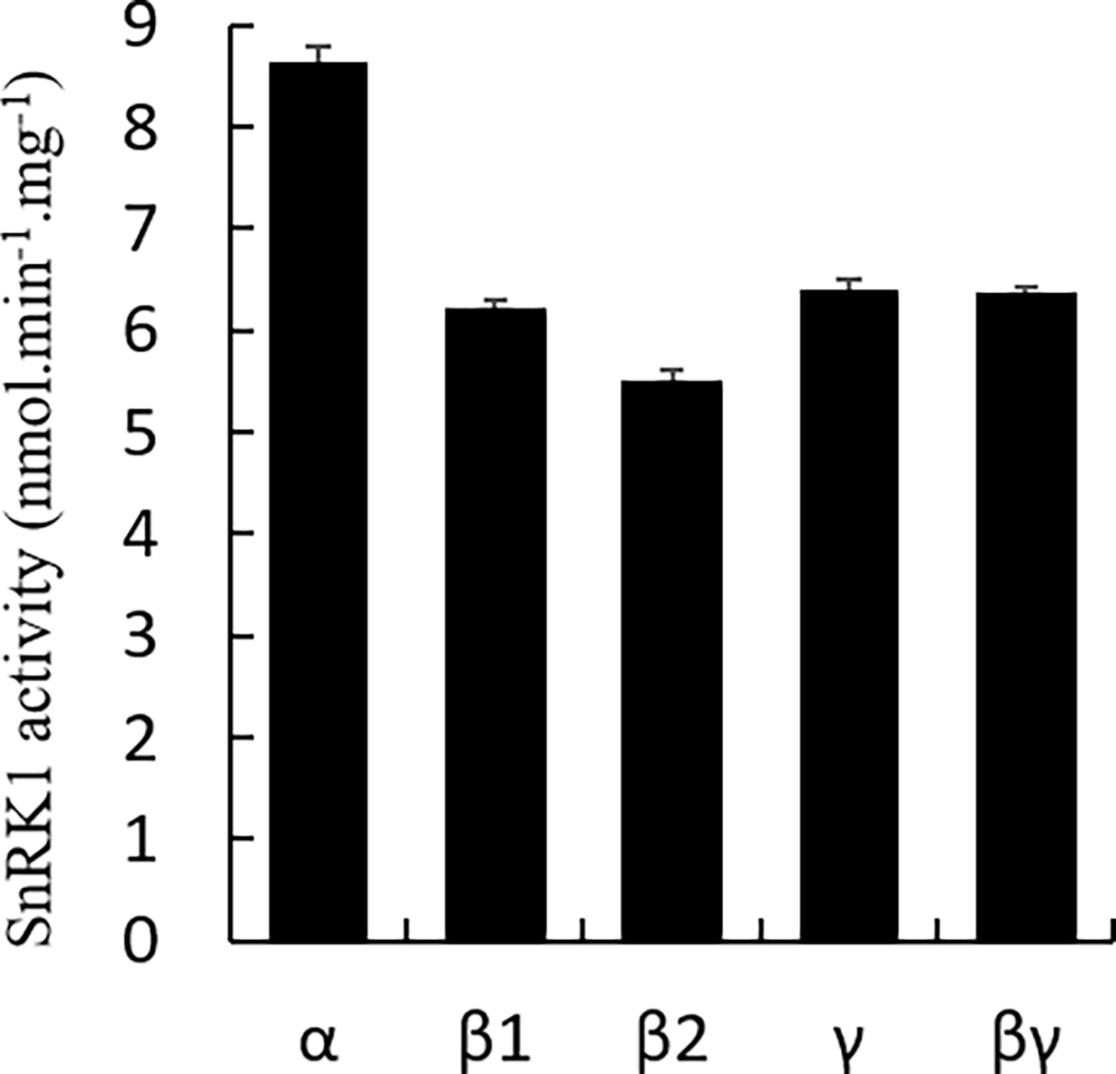
SnRK1 activity of purified protein from the overexpression transgenic plants of *StKIN*, *stKINβ1*, *stKINβ2*, *stKINγ* or *stKINβγ*. The total protein samples are from tubers of transgenic plants of *StKIN*, *stKINβ1*, *stKINβ2*, *stKINγ* or *stKINβγ*. During enzyme activity assay, boiled enzymes were used as negative control, the values reported on the graph are background-subtracted. Means of three independent samples are presented.

To investigate the functions of *stKIN*, *stKINβ1*, *stKINβ2*, *stKINγ* and *stKINβγ*, we developed OX transgenic lines of *stKIN*, *stKINβ1*, *stKINβ2*, *stKINγ* and *stKINβγ* fused to a HA tag sequence at the C-terminus of each gene, which expressed HA tagged stKIN, stKINβ1, stKINβ2, stKINγ and stKINβγ. We collected tubers from these OX transgenic lines and extracted the total protein. Then the protein samples were purified by Co-IP kit using a Co-IP kit to immuno-precipitate the stKIN, stKINβ1, stKINβ2, stKINγ and stKINβγ proteins. The purified protein samples were used for a SnRK1 activity assay, which showed highSnRK1 activity in all of the pellets of stKIN, stKINβ1, stKINβ2, stKINγ and stKINβγ (Fig 1), suggesting that all of these identified proteins are functional SnRK1-related proteins in potato tuber.

### Several different SnRK1 complexes are present in potato tuber

**Fig 2:**
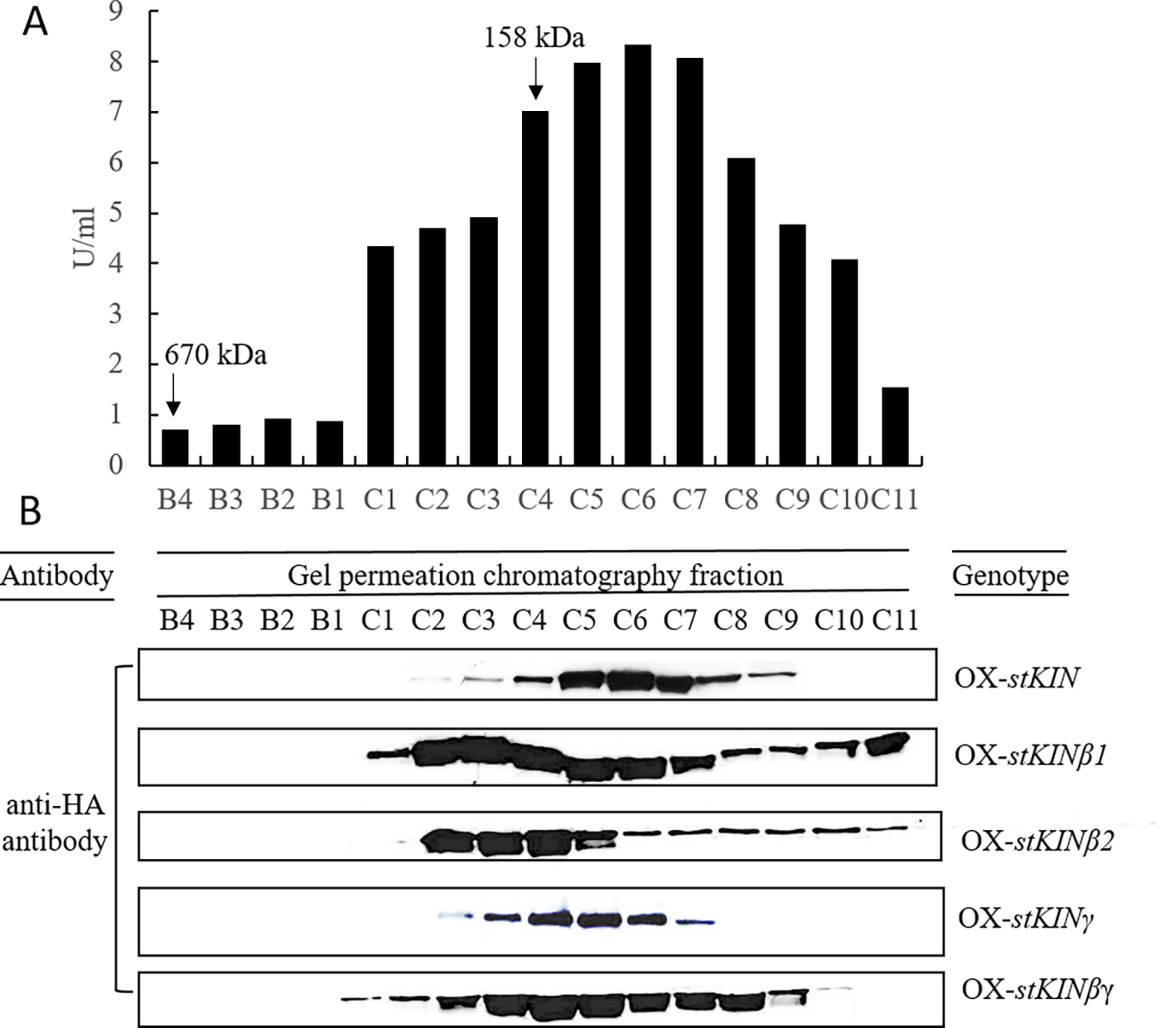
GPC analyses of transgenic tuber protein extracts. A) Activity distribution of SnRK1 from overexpression lines of *stKIN* of GPC fractions from B4 to C11. B) Proteins present in the indicated fractions from the GPC column were separated by SDS-PAGE and then probed with HA antibody in immunoblot analyses. The peak elution volumes of the molecular mass standards analyzed in the same GPC protocol are indicated at the top. The size of each fraction is 0.5ml, and the corresponding elution volume of B4 and C11 is 0.5 ml and 17.5, respectively.

In potato, we identified one α-subunit isoforms, along with two β- and two γ- isoforms, which may form four different possible SnRK1 isoenzymes. To collect additional evidence for possible isoforms of SnRK1 complex *in vivo*, we used gel permeation chromatography (GPC) to separate these isoforms into different fractions, as the sizes of different complexes might be different. The total protein from developing potato tubers of each of the OX transgenic lines of *stKIN*, *stKINβ1*, *stKINβ2*, *stKINγ and stKINβγ* were extracted separately in native buffer at 4°C. We then performed a partial purification of the SnRK1 complex by using anion-exchange chromatography (AEC) and GPC based on fast protein liquid chromatography (FPLC). The fractions were monitored by a kinase activity assay. We pooled all active fractions from the AEC and loaded the pooled sample on the GPC column. The first 40 fractions from the GPC were subjected to the kinase activity assay. We observed same enzyme activity distribution patterns among the 5 OX transgenic lines of *stKIN*, *stKINβ1*, *stKINβ2*, *stKINγ*, *stKINβγ* and wild type with high activity from fraction B4 to C11 that peaked at fractions C4 to C7. This observation suggested that overexpression would not affect the enzyme activity distribution. Compared with the standard curve, the active fractions were distributed between 670 kDa and 100 kDa, and the peak was located at approximately 120 kDa, while the fractions between 670 kDa and 120 kDa had high activity. The fractions from B4 to C11 for each of OX transgenic lines of *stKIN*, *stKINβ1*, *stKINβ2*, *stKINγ and stKINβγ* were used for western-blot to detect related protein by anti-HA antibody. These five subunits showed obviously different distribution profiles (Fig 2A and 2B). The stKIN protein was located primarily in C4 to C8, which was consistent with the activity peak. Both stKINβ1 and stKINβ2 were distributed in almost all active fractions; however, they showed different distribution patterns. The stKINβ1 protein was detected in fractions from C1 to C11 and was mainly located in fractions from C2 to C7 (580 kDa to 120 kDa). While stKINβ2 was detected in fraction from C2 to C11, and primarily located in fractions C2 to C5 (approximately or > 158 kDa). This suggested that they occurred in different SnRK1 complexes. The distribution pattern of stKINγ was primarily in fractions C4 to C6, similar to that of stKIN, while stKINβγ had a broader distribution from fractions from C1 to C10. However, in the higher activity fractions C1 to C3 and C8 to C10, we detected little stKINγ or stKINβγ (Fig 2A and 2B). Together, these data suggest that several different SnRK1 complexes exist in potato tuber during the tuber filling stage.

### Starch biosynthesis in overexpression and knockdown transgenic potato lines

**Fig 3:**
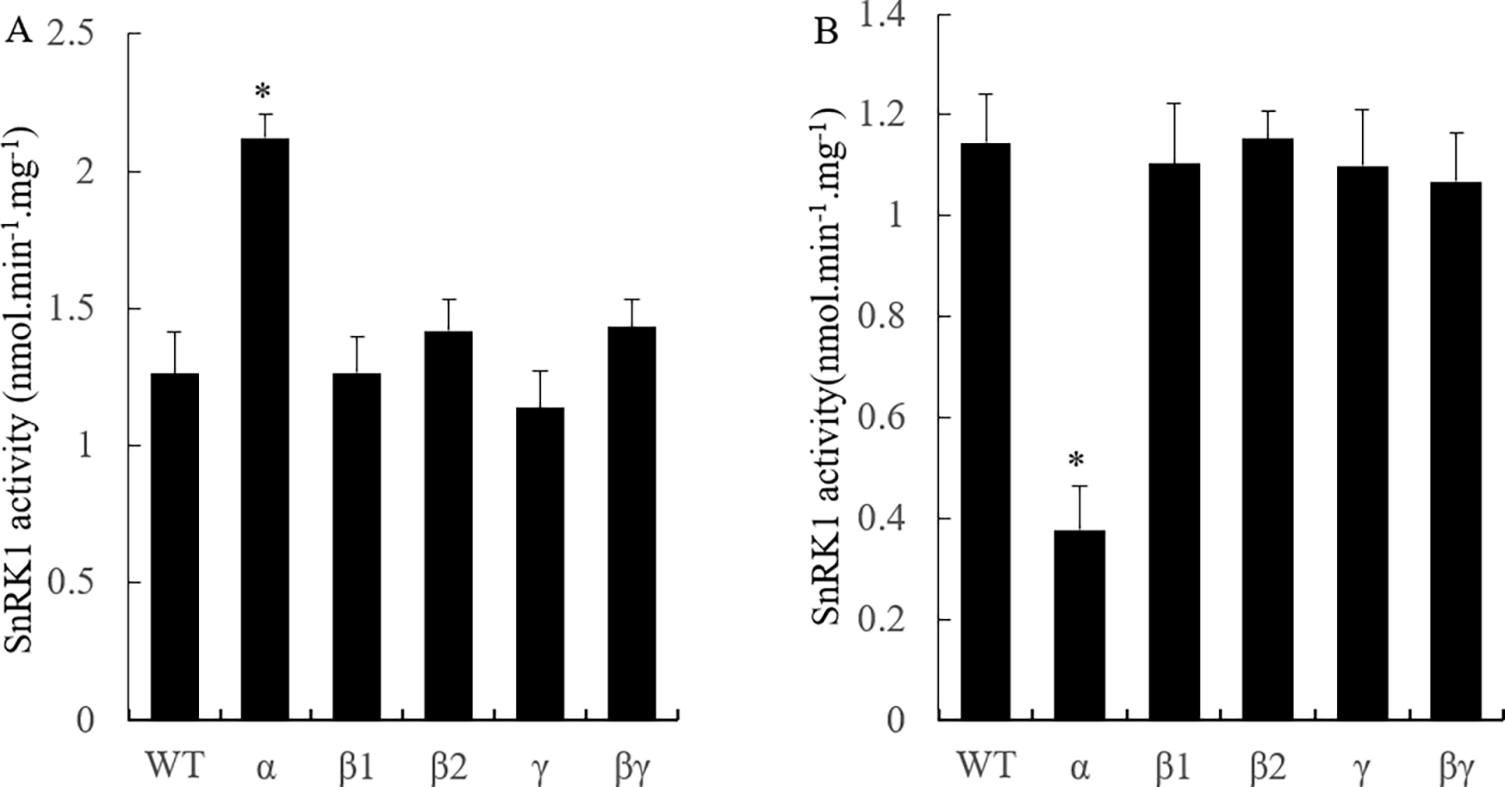
SnRK1 activity in total soluble extracts of potato tubers of transgenic plants and wild-type. (A) SnRK1 activity in the overexpression lines of *stKIN*, *stKINβ1*, *stKINβ2*, *stKINγ*, *stKINβγ* and wild-type control. (B) SnRK1 activity in the RNAi lines of *stKIN*, *stKINβ1*, *stKINβ2*, *stKINγ*, *stKINβγ* and wild-type control. Means of three independent samples are presented. The asterisk indicates a significant difference between means at *p* = 0.05.

**Fig 4:**
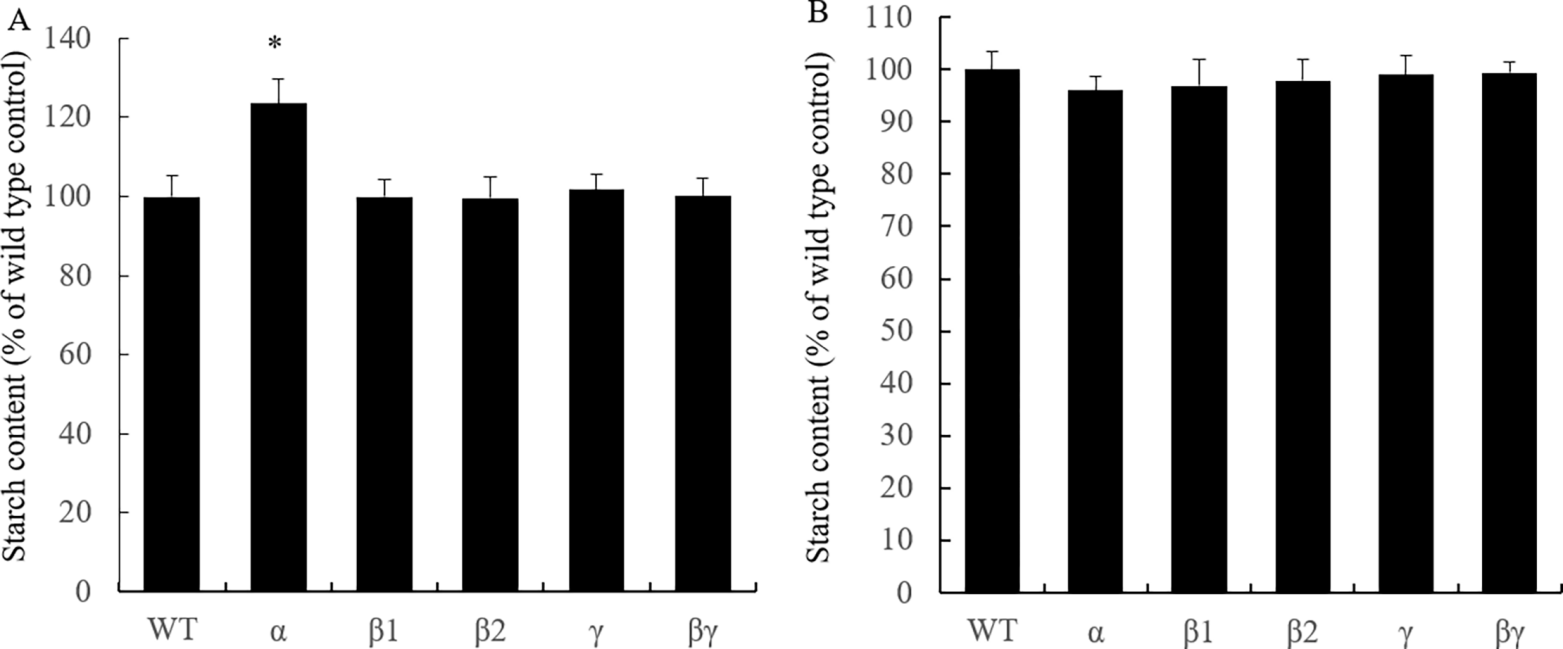
Starch content in total soluble extracts of potato tubers of transgenic plants and wild-type. (A) Starch content in overexpression lines the of *stKIN*, *stKINβ1*, *stKINβ2*, *stKINγ*, *stKINβγ* and wild-type control. (B) Starch content in the RNAi lines of *stKIN*, *stKINβ1*, *stKINβ2*, *stKINγ*, *stKINβγ* and wild-type control. Means of three independent samples are presented. The asterisk indicates a significant difference between means at *p* = 0.05.

To investigate tuber starch contents in the transgenic lines with altered SnRK1-related protein level, the tubers of transgenic lines were collected at 60 days after planting. In this case, we chose lines from three independent transformation events with the largest changes of transcript level for both OX and KD (transcript level decreases of >60% in the KD lines, and more than 2-fold transcript level increases in the OX lines). For each line, we randomly pooled three tubers to produce a homogenate, and the prepared samples were divided into 1 ml aliquots, which were used for enzyme assays and carbohydrate analyses. We measured SnRK1 activity in the KD and OX lines of *stKIN*, *stKINβ1*, *stKINβ2*, *stKINγ* and *stKINβγ*. Among the OX lines, only *stKIN* OX showed approximately a 40% increase in SnRK1 activity; we did not observe any changes in SnRK1 activity in the OX lines of *stKINβ1*, *stKINβ2*, *stKINγ* or *stKINβγ* (Fig 3A). Moreover, we observed approximately a 60% decrease in SnRK1 activity in all *stKIN* KD lines. However, we did not observe this effect in the KD lines of *stKINβ1*, *stKINβ2*, *stKINγ* and *stKINβγ* (Fig 3B). These data suggested that *stKIN* is the primary SnRK1 enzyme. We further determined the starch content in the tubers of the transgenic lines of SnRK1-related genes. We observed significantly changes in the starch content in both the OX and KD lines of *stKIN*, including approximate 25% starch content increase in the OX transgenic lines and no change in starch content in the KD lines (Fig 4A and 4B). The starch content increase in OX transgenic lines was consistent with the results of a previous study, which showed a starch content increase under *stKIN* OX [7]. In addition, we found no significant changes in starch content in either the KD or OX lines of *stKINβ1*, *stKINβ2*, *stKINγ or stKINβγ* (Fig 4A and 4B).

## Discussion

For the SnRK1 complex purification, based on the protein standards, the sizes of fractions B4 to C4 were between around 670Kda and 158Kda, and the size of protein fractions C5 to C11 were located in 158 to 120 kDa. This result indicated that the enzyme complexes found in fractions B4 to C3 were different from those in fractions C4 to C11. It might be because of that SnRK1 complexes interact with other proteins to form high-order regulatory complex in fractions B4 to C3. For examples, in potato, SnRK1 was proved to form a protein complex with an invertase and invertase inhibitor to regulate the invertase activity in potato tubers [17]; in rice, SnRK1A-interacting negative regulator 1 proteins (SKINs) modulate nutrient starvation signaling by antagonizing the function of SnRK1 [18].

When analyzing the SnRK1 activity in the KD transgenic lines of *stKIN*, we found that SnRK1 activity decrease by more than 70%. In addition, we observed high activity in fractions B4 to C1 and C10 to C11, but we did not detect any stKIN protein, indicating that the stKIN is the primary SnRK1 α subunit in potato; however, other SnRK1 isoforms are likely present but not identified. We observed 25% starch content increase in OX transgenic lines of *stKIN*, however, no such any changes in starch content KD transgenic lines of *stKIN*, indicating that other regulatory pathways controlling carbohydrate metabolism are present in potato tuber. There are no such changes were found in the transgenic lines of *stKINβ1*, *stKINβ2*, *stKINγ* or *stKINβγ*, indicating that other kinds of such subunits might compensate for the functions of *stKINβ1*, *stKINβ2*, *stKINγ* or *stKINβγ*. From the distribution of beta subunits in the fractions, we can see that both stKINβ1 and stKINβ2 beta subunits are broadly distributed in most of fractions, which suggested the importance of these two beta subunits. The importance of beta subunits has been proven in other species. Lopez-Paz *et al*. (2009) reported that ZmKIN_β_ stabilize the AKIN_βγ_/SnRK1/AKIN_β_ complex [5]_._ Moreover, when *AKIN10* and *AKINβγ* were co-expressed in mammal cells, an antibody to AKINβγ could not co-precipitate AKIN10, while when AKIN10, AKINβ1 and AKINβγ were co-expressed, the AKINβγ antibody could co-precipitate both AKIN10 and AKINβ1[6]. These data indicate that AKINβ1 is important to form a tetrameric complex.

In summary, a few different isoforms of SnRK1 are present in potato tuber. We identified five SnRK1-related proteins, and the overexpression of stKIN affects starch content.

## Supporting information

S1 File Uncropped and un-altered western blot image.pptx. (PPTX)
