# Supplementary material for: Identification of multiple genes encoding SnRK1 subunits in potato tuber
Source: PLoS One. 2018 Jul 6;13(7):e0200321. doi: 10.1371/journal.pone.0200321 (PMC6034879; doi:10.1371/journal.pone.0200321)
Supplement: S1 File — (PPTX) [file pone.0200321.s001.pptx]

## Slide 1
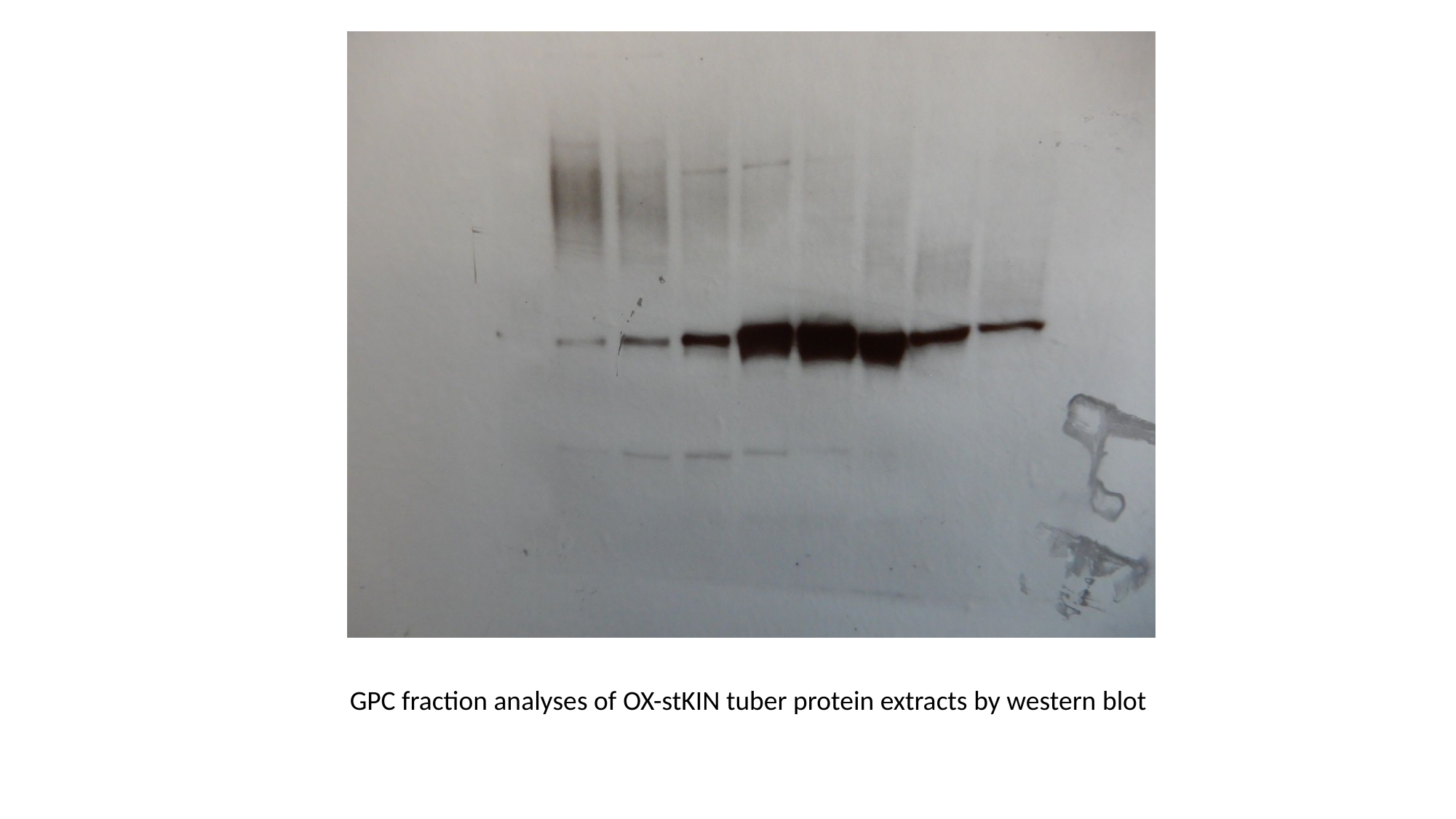

GPC fraction analyses of OX-stKIN tuber protein extracts by western blot

## Slide 2
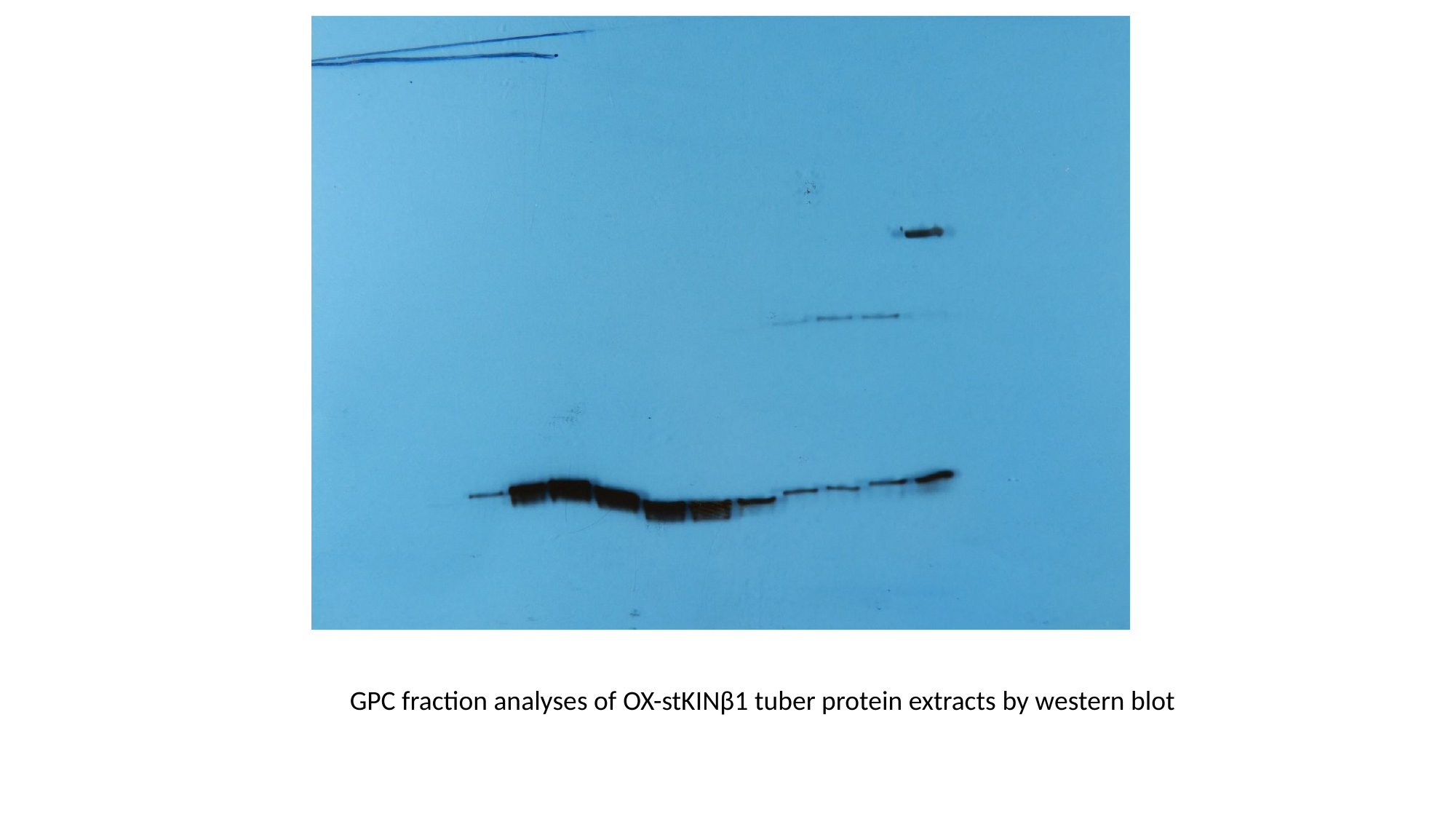

GPC fraction analyses of OX-stKINβ1 tuber protein extracts by western blot

## Slide 3
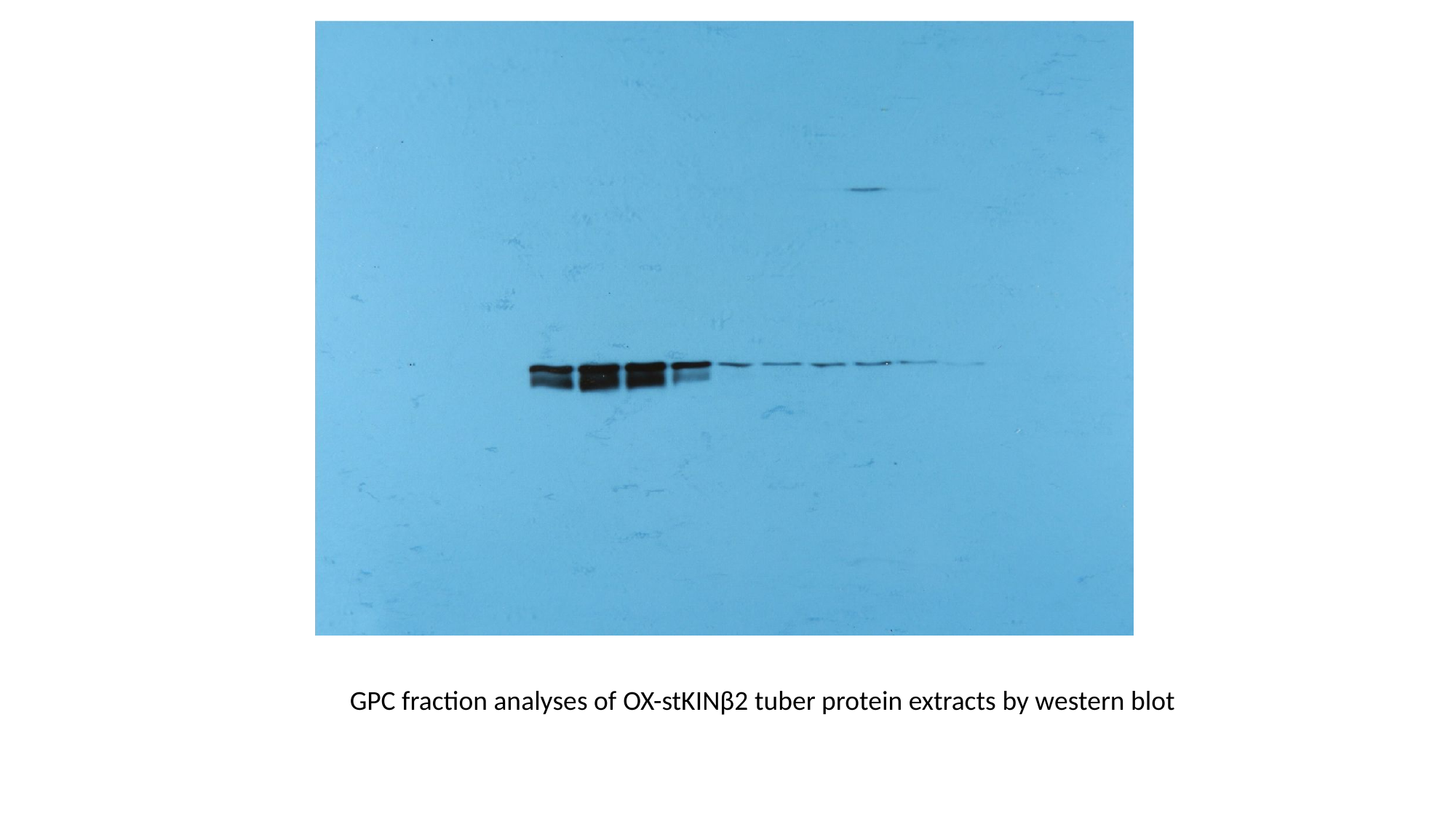

GPC fraction analyses of OX-stKINβ2 tuber protein extracts by western blot

## Slide 4
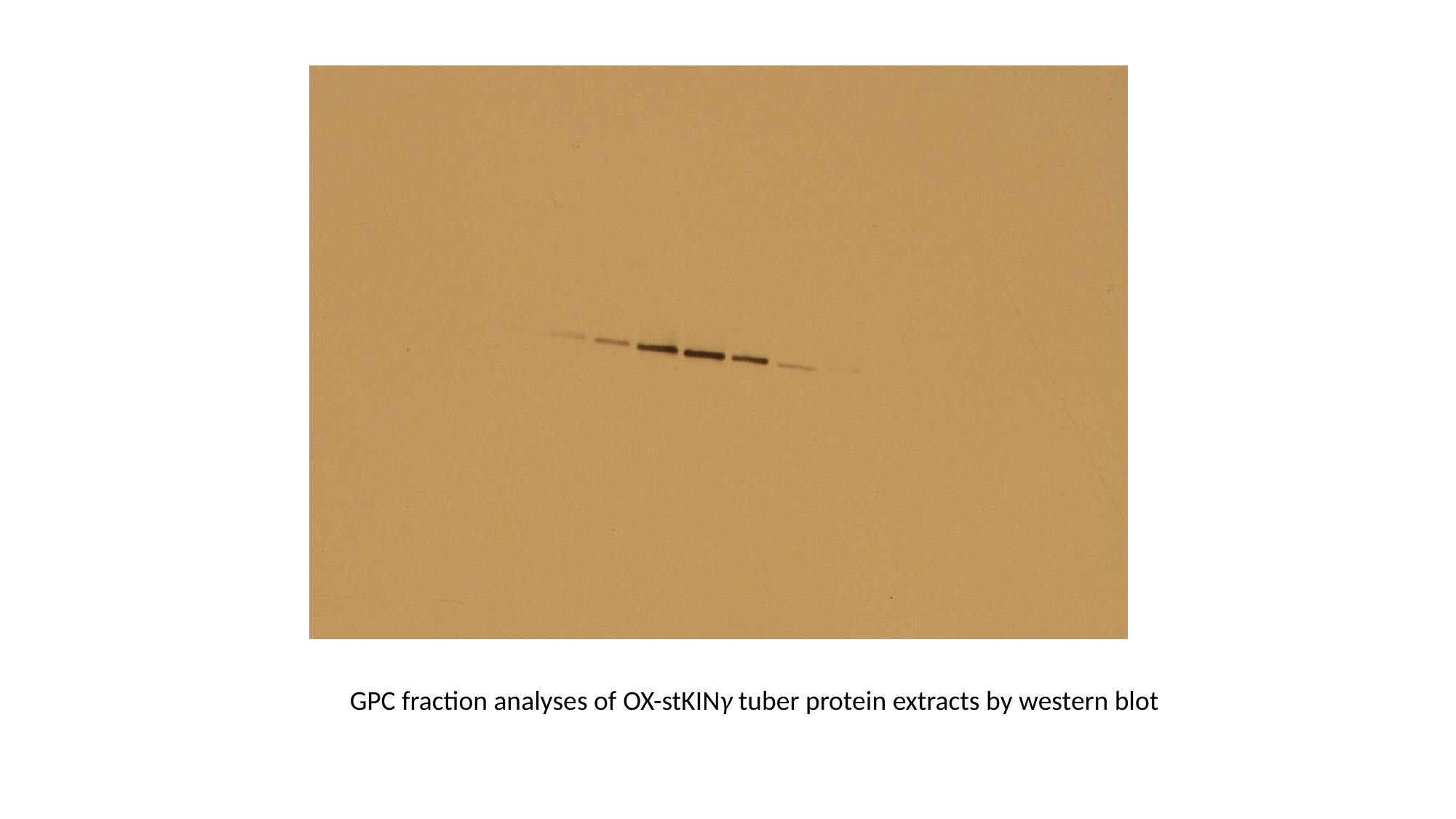

GPC fraction analyses of OX-stKINγ tuber protein extracts by western blot

## Slide 5
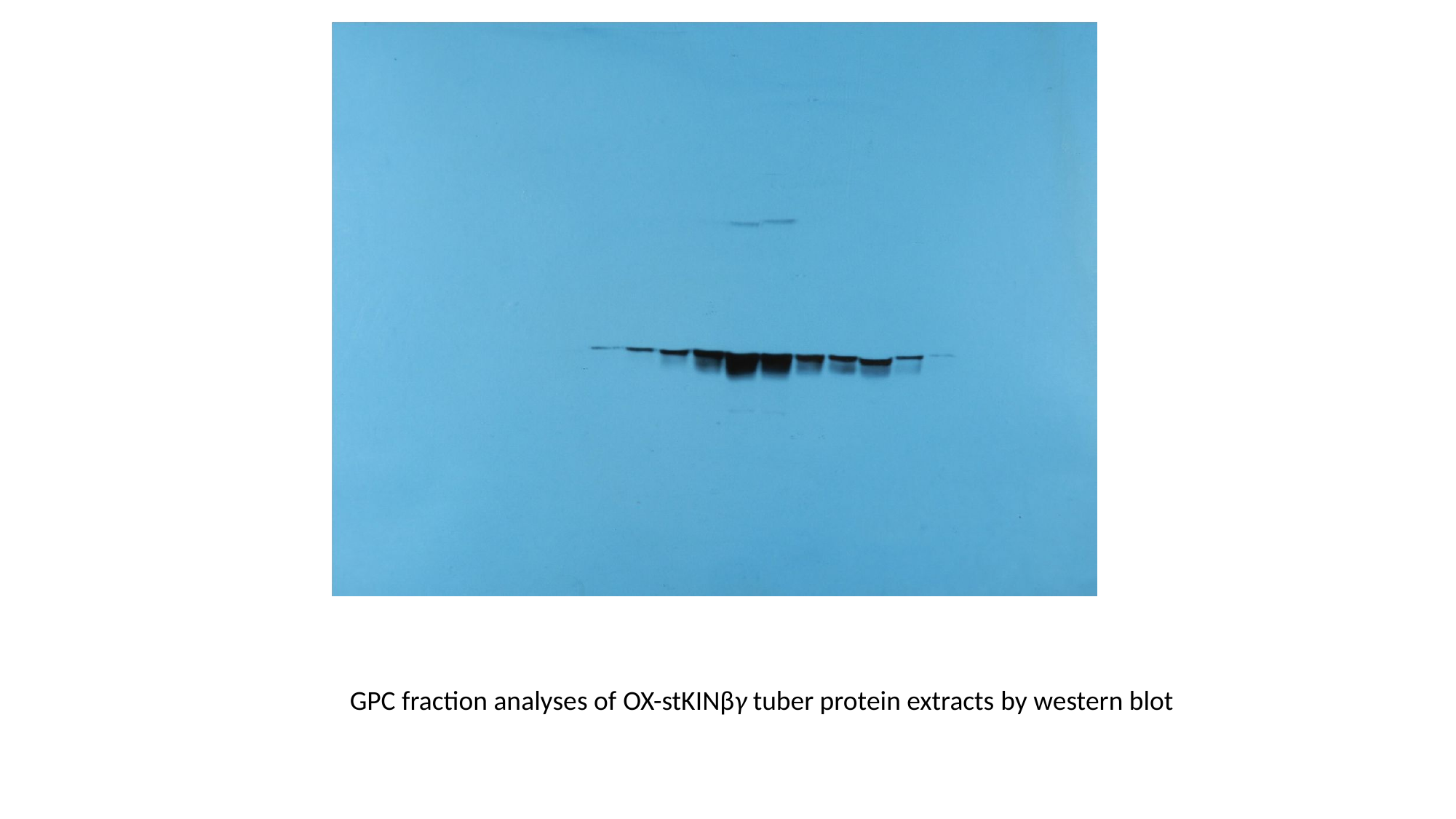

GPC fraction analyses of OX-stKINβγ tuber protein extracts by western blot
